# Patent ductus arteriosus in Qatar: a retrospective study on epidemiology and neonatal outcomes following transcatheter closure

**DOI:** 10.3389/fped.2026.1798842

**Published:** 2026-06-26

**Authors:** Safaa Elmoh, Hala Fouad, Ihsan Elhalabi, Soha Roger Dargham, Mange Manyama, Samir Gupta, Hesham Al-Saloos

**Affiliations:** 1 Division of Medical Education, Weill Cornell Medicine—Qatar, Doha, Qatar; 2 Division of Cardiology, Sidra Medicine, Doha, Qatar; 3 Division of Neonatology, Department of Pediatrics, Sidra Medicine, Doha, Qatar

**Keywords:** congenital heart disease, outcomes, patent ductus arteriosus, prematurity, transcatheter device closure

## Abstract

**Introduction:**

Patent ductus arteriosus (PDA) is a congenital heart defect resulting from failure of the ductus arteriosus to close after birth. It is particularly common in preterm infants and may become hemodynamically significant, requiring intervention. Transcatheter device closure offers a minimally invasive treatment option, though it can be technically challenging in this population.

**Methods:**

This retrospective study included 80 infants diagnosed with PDA at Sidra Medicine, Qatar, between 2018 and 2024. Data were obtained from electronic health records. Patients who underwent medical treatment or transcatheter device closure were included, while those requiring surgical intervention were excluded. Variables collected included gestational age, birth weight, comorbidities, and PDA characteristics. Outcomes assessed were implant success and complications. Statistical analysis was performed using SPSS version 29, with significance set at *p* ≤ 0.05.

**Results:**

Of the 80 patients, 60% were male and 83.75% were born prematurely (<37 weeks gestation). Transcatheter device closure was performed in 52 patients (65%). Gestational age <37 weeks was significantly associated with catheter-based treatment (*p* ≤ 0.05). The presence of a PDA murmur increased the likelihood of catheter-based intervention eightfold (OR 8.125; *p* < 0.001), and larger PDA diameter was also a significant predictor (OR 4.628; *p* < 0.001). Among those undergoing transcatheter closure, implant success was achieved in 45 of 52 patients (86.5%) on the first attempt and in all patients (100%) after repeat intervention. Intraprocedural complications included device embolization in 4 patients. Residual shunt was observed immediately post-procedure in 11 patients (21.2%) but resolved in all 48 patients with six-month follow-up data. Time to discharge correlated negatively with gestational age (R =–0.365; *p* = 0.014) and birth weight (R =–0.383; *p* = 0.011). Non-procedure-related mortality occurred in 5 patients (9.6%).

**Discussion:**

PDA closure demonstrates high procedural success and acceptable safety in preterm infants. Key factors influencing intervention and outcomes include gestational age, birth weight, and ductal size. These findings support the feasibility of transcatheter approaches in this vulnerable population, while highlighting the importance of patient selection and clinical characteristics in predicting outcomes and hospital course.

## Introduction

Patent ductus arteriosus (PDA) is a congenital heart defect characterized by the failure of the ductus arteriosus (DA) to close within 72 h after birth. In utero, the DA plays a critical role in fetal circulation, connecting the pulmonary artery to the aorta and allowing oxygenated blood from the placenta to bypass the non-functioning lungs (1). Following full-term birth, physiological changes, including increased oxygen tension and decreased prostaglandin levels, typically lead to DA constriction within 24–72 h, with complete anatomical closure occurring by 2–3 weeks of life (2). However, when this process fails, in otherwise normal heart structure, PDA results in a persistent left-to-right shunt, causing increased pulmonary blood flow and potential complications such as pulmonary hypertension, heart failure, and end-organ damage.

The incidence of PDA varies significantly between term and preterm infants, affecting approximately 5%–10% of term infants and 20%–60% of preterm infants (3). The condition is particularly prevalent in low birth weight and extremely preterm neonates, with rates as high as 80% in infants born before 28 weeks of gestation (4). Risk factors for PDA include prematurity, maternal diabetes mellitus, maternal infections (e.g., rubella, chorioamnionitis), and genetic syndromes such as trisomy 21 and Noonan syndrome (5–7). Clinical presentation depends on the size of the defect, with larger, persistent PDAs cause more pronounced symptoms, including a continuous “machine-like” murmur, tachycardia, bounding peripheral pulses, and widened pulse pressure (2). Left untreated, PDA can lead to significant morbidity, including bronchopulmonary dysplasia, necrotizing enterocolitis (NEC), intraventricular hemorrhage, and congestive heart failure.

Management strategies for PDA range from conservative approaches to pharmacological interventions and invasive procedures. Pharmacological treatments, including indomethacin, ibuprofen, and acetaminophen, are commonly used as first-line therapies, particularly in preterm infants. However, these treatments are less effective beyond the third week of life and are associated with a 25% rate of PDA reopening (8). For hemodynamically significant persistent PDAs, surgical ligation or transcatheter closure is often required (3). Transcatheter closure, first introduced in 1967, has evolved significantly with advancements in device technology, such as the Amplatzer Duct Occluder and its variants (9–11). This minimally invasive procedure is now widely used, particularly in term infants, with success rates exceeding 98% and fewer complications compared to surgical ligation (12, 13). However, its application in preterm and low birth weight infants remains limited due to higher risks of adverse events, including device embolization and vascular injury. Recent meta-analytic evidence comparing active treatment and expectant management has further highlighted the complexity of decision-making in this population (14).

The data on the epidemiology of PDA and the outcomes of transcatheter closure in Qatar are scarce. Understanding the burden of PDA in Qatar is critical for improving neonatal outcomes and informing future research priorities. This study aimed to address these gaps by providing a detailed analysis of PDA epidemiology and the outcomes of transcatheter closure in neonates with PDA in Qatar. Through a retrospective review, we evaluated procedural success rates, complications, and factors influencing outcomes, including patient demographics and PDA characteristics.

## Materials and methods

This retrospective study analyzed infants with PDA at Sidra Medicine, Qatar. Sidra Medicine is a tertiary referral center that manages predominantly complex cases, whereas less complex PDA cases receiving medical therapy are treated at secondary centers. The data was gathered from patients’ electronic health records and analyzed. The study included patients who were diagnosed with PDA between 2018 and 2024 and received either medical treatment or transcatheter device closure. Patients with complex cardiac comorbidities requiring surgical intervention were excluded. Demographic data collected included sex, ethnicity, gestational age at birth, birth weight, comorbidities, maternal risk factors and PDA characteristics (e.g., type and diameter). All transcatheter closures were performed using the Amplatzer Piccolo Occluder. Device size selection was based on ductal morphology and minimal ductal diameter, following manufacturer recommendations and operator discretion.

Procedural dates and discharge information were recorded to assess outcomes. Outcomes were categorized as immediate (intra-procedural), early post-procedural (prior to discharge), and six-month follow-up outcomes. Analyses were performed using complete-case data for each variable. Immediate outcomes included implant success and complications such as device embolization, left pulmonary artery obstruction, and residual shunt. Tricuspid regurgitation severity was documented before and after the procedure; it was analyzed as an echocardiographic finding rather than assumed to represent a procedural complication. Long-term outcomes, such as residual shunt or other complications within six months, were also documented. One patient in the transcatheter closure group was excluded from outcome evaluation due to substantial missing procedural and follow-up data.

Implant success was defined as successful deployment of the occlusion device in the intended anatomical position with stable placement and without the need for surgical conversion. Residual shunt and other procedural complications were recorded and analyzed as separate outcomes.

Data analysis was carried out using IBM SPSS (version 29, Armonk, NY, USA). Gestational age at birth was categorized into ≤31 weeks, 32–36 weeks, and ≥37 weeks. Continuous variables, including age at transcatheter device closure, time to discharge, and birth weight, were summarized using means and standard deviations where normally distributed. Data distribution was assessed using the Shapiro–Wilk test and visual inspection of histograms. Continuous variables approximating normal distribution were analyzed using Pearson’s correlation coefficient; otherwise, Spearman’s rank correlation was applied. Categorical variables were compared using Chi-square tests.

Univariable logistic regression analysis was performed to estimate odds ratios and 95% confidence intervals for associations between clinical variables and treatment allocation. Missing data were handled using complete-case analysis, and denominators therefore vary across specific analyses based on data availability.

Given the exploratory nature of the analyses and the limited sample size, multivariable adjustment was not performed to avoid overfitting. No correction for multiple comparisons was applied, and reported *p*-values should be interpreted as nominal. A p-value ≤ 0.05 was considered statistically significant.

## Results

### Patient demographics

Of the 80 patients included in this study, the majority (60%) were male (Table 1). Among the patients, majority (83.75%) were born premature (gestational age <37 weeks), while 16.25% were born at term (≥37 weeks). Patients’ nationalities were classified into MENA (Middle East and North Africa) and non-MENA regions, based on UNICEF’s 2021 definition of MENA countries, which include Algeria, Bahrain, Djibouti, Egypt, Iran, Iraq, Jordan, Kuwait, Lebanon, Libya, Morocco, Oman, Palestine, Qatar, Saudi Arabia, Sudan, Syria, Tunisia, the United Arab Emirates, and Yemen; a total of 47 (58.75%) patients were from the MENA region. In our sample, 27 patients (33.75%) were managed medically, while 53 patients (66.25%) ultimately underwent transcatheter device closure. Group classification was based on final management strategy. Some patients in the transcatheter group may have received prior pharmacologic therapy before proceeding to device closure, reflecting real-world clinical practice.

**Table 1:** Demographics of patients with PDA.

Parameter	*n* (%)
Sex	
Male	48/80 (60%)
Female	32/80 (40%)
Region of origin	
MENA region	47/80 (58.75%)
Non-MENA region	33/80 (41.25%)
Gestation age at birth	
Term (≥37 weeks)	13/80 (16.25%)
Preterm (<37 weeks)	67/80 (83.75%)
PDA subtypes	
F-subtype	13/30
C-subtype	11/30
A-subtype	3/30
Other subtypes	3/30
Treatment modality	
Medical	27/80 (33.75%)
Transcatheter device	53/80 (66.25%)

Data are presented as *n* (%) unless otherwise specified. Percentages are calculated using available cases per variable.

### The relationship between neonatal risk factors, comorbidities and PDA treatment approach

This study also examined risk factors and comorbidities in patients with patent ductus arteriosus and their correlation with treatment selection decisions (Table 2). Some patients had incomplete records for certain variables, resulting in number of cases less than the overall sample size of 80 for some categories. Prematurity and maternal diabetes mellitus were among the risk factors identified in this sample. Further analysis revealed a significant association between gestational age at birth and the choice of PDA management method (*p* ≤ 0.05). Specifically, patients born at less than thirty-seven weeks of gestation were more likely to undergo catheter-based treatment rather than medical treatment.

**Table 2:** Association between neonatal risk factors, comorbidities, and PDA treatment approach.

Risk factor/comorbidity	Medical treatment (*n*)	Transcatheter closure (*n*)	*P*-value
Gestation age at birth			
Term	0/77	10/77	0.006
Preterm	27/77	40/77	
Maternal Diabetes Mellitus			0.540
No	12/58	29/58	
Yes	7/58	10/58	
Patent foramen ovale			0.126
No	5/77	18/77	
Yes	22/77	32/77	
Respiratory distress syndrome			0.327
No	8/76	21/76	
Yes	19/76	28/76	
Necrotizing enterocolitis			0.066
No	14/78	41/78	
Yes	11/78	12/78	
Pulmonary hypertension			0.714
No	23/75	43/75	
Yes	4/75	5/75	
Bronchopulmonary dysplasia			0.309
No	25/76	40/76	
Yes	2/76	9/76	
Mean PDA diameter (mm)	2.55	3.69	<0.001
Presence of PDA murmur			<0.001
No	13/75	8/75	
Yes	9/75	45/75	
Tricuspid regurgitation*			<0.001
No	6/75	33/75	
Yes	20/75	16/75	

* TR was analyzed as an echocardiographic finding and not classified as a procedural complication.

Observed discrepancies in denominators are attributable to variable-specific differences in the number of cases with complete data.

Associated clinical conditions observed in patients with PDA included respiratory distress syndrome, necrotizing enterocolitis, pulmonary hypertension, and patent foramen ovale. In this retrospective analysis, no statistically significant association was identified between these conditions and the final management strategy. However, this finding does not exclude their potential role in individual clinical decision-making.

Our findings also revealed that the presence of a PDA murmur was associated with an 8-fold increased likelihood of undergoing catheter-based treatment [OR 8.125; 95% CI (2.612–25.274); *P* < 0.001] (Table 2). Additionally, a larger PDA diameter correlated with a higher likelihood of catheter-based treatment [OR 4.628; 95% CI (2.211–9.689); *P* < 0.001].

### Outcomes in patients with PDA who underwent transcatheter device closure

Table 3 summarizes the demographics of patients who underwent transcatheter device closure procedure. Denominators vary across specific analyses due to missing data for certain variables; the applicable denominator is reported for each outcome. Of the 53 patients who underwent transcatheter device closure, 52 had sufficient procedural and follow-up data available for outcome analysis. Among patients who underwent transcatheter closure, 45 had documented gestational age at birth, and 30 had available data on PDA subtype classification.

**Table 3:** Demographic characteristics of patients with PDA who underwent transcatheter device closure.

Parameter	N* (%)
Sex	
Male	32/52 (61.5)
Female	20/52 (38.5)
Gestation age at birth	
≤31 weeks	31/45 (68.9)
32–36 weeks	5/45 (11.1)
≥37 weeks	9/45 (20)
PDA subtypes	
F-subtype	13/30 (43.3)
C-subtype	11/30 (36.7)
A-subtype	3/30 (10)
Other subtypes	3/30 (10)
Age at procedure	Mean ± SD = 58 ± 31.9 days
Birth weight	Mean ± SD = 1330.6 ± 941.8 g
PDA diameter	Mean ± SD = 3.7 ± 0.8

* Outcome analyses were performed on 52 patients with complete data.

* Observed discrepancies in denominators are attributable to variable-specific differences in the number of cases with complete data.

About 80% of the patients were born prematurely (<37 weeks). Among the 30 patients with available angiographic data, F-subtype and C-subtype were the most frequently documented (Table 3) according to established angiographic classifications (15, 16). Given the incomplete subtype data, these findings should be interpreted as descriptive rather than representative of the entire cohort.

The patients’ ages at the time of the procedure ranged from 24 to 93 days, with an average age of 58 ± 31.9 days. The mean birth weight was 1330.6 ± 941.8 grams, and the mean diameter of the PDA was 3.7 ± 0.8 mm.

**Figure 1:**
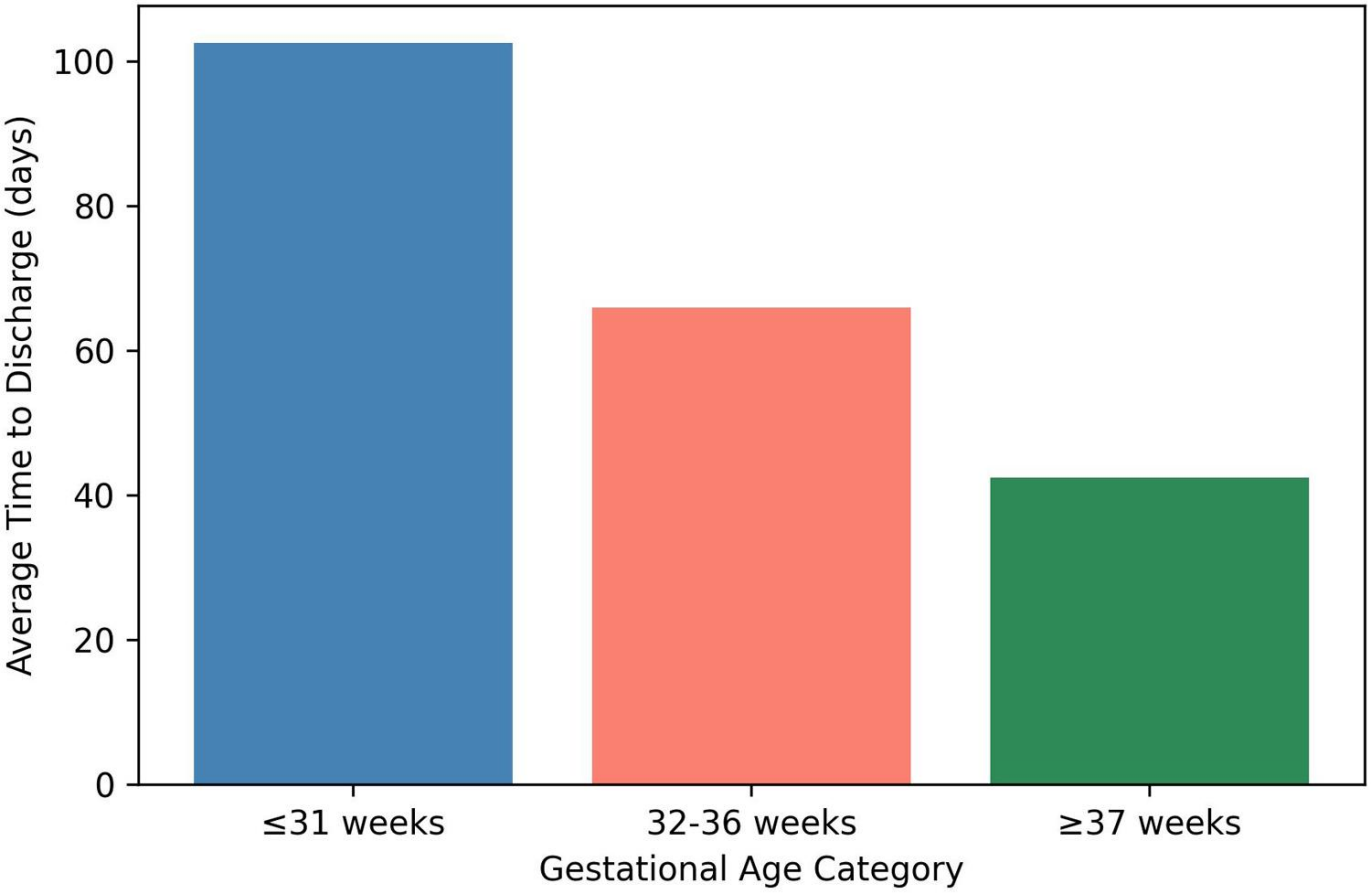
Time to discharge according to gestational age at birth. Bars represent mean (±SD) length of hospital stay (days) for ≤31 weeks (*n* = 102.6), 32–36 weeks (*n* = 66), and ≥37 weeks (*n* = 42.4). Pearson correlation demonstrated a significant association between gestational age and length of stay (*p* = 0.014).

The mean time to discharge for term-born patients was 42.4 days, while the most premature patients had the longest mean time to discharge at 102.6 days (Figure 1). Implant success, defined as successful device placement on the initial attempt, was achieved in 45/52 patients (86.5%) (Table 4). All patients with an unsuccessful initial procedure underwent a repeat intervention within 24 h, achieving a 100% success rate on the second attempt. Intraprocedural device embolization occurred in 7.7% (*n* = 4) of patients. Of the four patients who experienced intraprocedural device embolization (one in each pulmonary artery), a secondary device closure procedure was required. In the remaining two patients, the medical records documented embolization that subsequently resolved without surgical intervention; however, detailed procedural descriptions regarding the mechanism of resolution were not available in the charts.

**Table 4:** Outcomes in patients who underwent transcatheter device closure.

Outcome	*N*	Rate (%)
Intra-procedural		
Intra-procedural device embolization	4/52	7.7%
Other intra-procedural complications	14/52	26.9%
Immediate post-procedure		
Implant success	45/52	86.5%
Residual shunt post-procedure	11/52	21.2%
Post-procedural obstruction of the left pulmonary artery	4/52	7.7%
Tricuspid regurgitation*	29/52	55.8%
At 6-months		
Residual shunt at 6 months	0/48	0.0%
Other adverse outcomes within 6 months	15/52	28.8%

Outcome analyses were performed on 52 patients with complete data.

* TR was analyzed as an echocardiographic finding and not classified as a procedural complication.

The denominator for residual shunt at 6 months was 48, reflecting 4 cases lost to follow-up.

Immediate post-procedure outcomes, such as residual shunt and left pulmonary artery obstruction, were noted in 11/52 patients (21.2%) and 4/52 patients (7.7%), respectively (Table 4). No patient exhibited a residual shunt at 6 months follow up; however, 4 patients did not have this information recorded in their electronic health records. All 4 of these patients showed no evidence of a residual shunt during their hospital stay post-procedure.

Significant tricuspid regurgitation (TR), defined as mild or greater, was identified in 9 patients (25%) of the 36 patients with documented pre-procedural TR. Notably, the high rate of tricuspid regurgitation is more likely to reflect pre-existing tricuspid regurgitation rather than an outcome of the device closure procedure itself, as the condition is commonly present in infants. Tricuspid regurgitation was documented based on echocardiographic reports and analyzed as an existing cardiac finding rather than as a procedural complication. TR severity (mild or greater) was recorded pre-procedure and at post-procedural assessment when available. Denominators vary based on data availability and timing of assessment. Thus, pre-procedural TR was documented among 75 patients, whereas post-procedural TR data were available for 52 patients.

Five of 52 patients (9.6%) died within 180 days post-procedure and all deaths were not procedure related due to extreme prematurity or other systemic illness.

Additional analysis was performed to examine the effect of PDA diameter on procedural outcomes. A significant difference was observed in two outcomes: infants who experienced intraprocedural device embolization had a smaller mean PDA diameter compared with those who did not (*p* = 0.037). Conversely, patients with a residual shunt after the procedure tended to have a larger mean PDA diameter (*p* = 0.035).

### Impact of prematurity and birth weight on outcomes of transcatheter device closure

We conducted further analysis to determine the relationship between gestational age at birth and several outcomes of transcatheter device closure. Secondary analysis using Pearson’s correlation revealed a significant association between time to discharge and gestational age at birth (R = −0.365; *p* = 0.014), indicating that more premature patients (≤31 weeks) had longer discharge times (Table 5). The chi-squared test revealed that only implant success showed a significant association with gestational age at birth, with higher success rates observed among premature patients.

**Table 5:** Relationship between gestational age at birth and procedural outcomes.

Outcome	≤31 weeks	32–36 weeks	≥37 weeks	P-value
Implant success	35/45	3/45	7/45	0.032
Intra-procedural device embolization	2/4	0/4	2/4	0.217
Other intra-procedural complications	9/14	1/14	4/14	0.604
Residual shunt post-procedure	8/14	2/14	1/14	0.322
Post-procedural obstruction of the left pulmonary artery	2/4	2/4	0/4	0.052
Tricuspid regurgitation	20/29	2/29	7/29	0.543
Time to discharge (days)	102.6	66	42.4	0.014

Our analysis examined the relationship between birth weight and time to discharge. Results revealed a significant inverse correlation (*r* = −0.383, *p* = 0.011), indicating that lower birth weight was associated with longer hospital stays before discharge.

## Discussion

The management of patent ductus arteriosus in preterm infants shows considerable clinical variation across different medical settings. Recent trends have moved away from aggressive closure strategies toward a more conservative approach that allows for spontaneous closure, potentially eliminating the need for medical intervention. This research examined the epidemiological patterns of PDA and outcomes following transcatheter device closure in Qatar’s patient population. By analyzing local outcomes, this study aims to enhance patient care protocols considering the evolving nature of the procedure and the variability in success rates observed across different populations and healthcare settings. Given the modest sample size and lack of multivariable adjustment, the associations reported should be interpreted as exploratory and hypothesis-generating rather than causal.

Most patients in this study were male and born prematurely. The higher occurrence of PDA in preterm infants is linked to the immaturity of normal closure mechanisms (2). Non-structural cardiac comorbidities observed in patients with PDA in this study include respiratory distress syndrome, necrotizing enterocolitis, and pulmonary hypertension. The choice of PDA treatment method, however, was not influenced by the presence of these conditions. Similar comorbidities have been reported in other studies, albeit with varying prevalence (17–19). The development of NEC in PDA patients is linked to flow reversal in the abdominal aorta and reduced mesenteric blood flow velocity, which impair intestinal perfusion. Pulmonary hypertension results from the left-to-right shunt in PDA, which increases blood flow to the pulmonary vessels, leading to pulmonary vascular remodeling, elevated pulmonary vascular resistance, and eventual pulmonary hypertension (20).

Our study also found that patients with a PDA murmur were 8 times more likely to undergo transcatheter device closure, supporting current clinical guidelines. These guidelines classify the presence of a murmur, along with indicators such as tachycardia, as criteria for hemodynamically significant PDA and provide a Class IIa recommendation for transcatheter occlusion (21, 22). Larger PDA diameters increased the likelihood of requiring transcatheter device closure intervention. This finding is consistent with previous studies that have established a correlation between PDA size and treatment efficacy, with larger PDAs being more resistant to closure via indomethacin therapy, while smaller PDAs demonstrate higher success rates with acetaminophen (23, 24). Due to the relatively small sample size, multivariable adjustment was not performed to prevent overfitting and unstable estimates, despite clinical factors such as gestational age, birth weight, and PDA diameter influencing treatment allocation. Accordingly, comparisons between treatment groups should be interpreted as descriptive rather than causal.

Our analysis of transcatheter device closure outcomes in 52 PDA patients, predominantly preterm infants (80.0%), showed an 86.5% success rate on the initial attempt, reaching 100% after second attempts. Although prematurity has historically been associated with conservative or medical management, recent advances in device technology have expanded the feasibility of transcatheter closure in selected preterm and low-birth-weight infants (25, 26). As a tertiary referral center managing more complex cases, our cohort may reflect referral bias, with preterm infants requiring intervention more likely to be referred for catheter-based closure. Therefore, this finding should be interpreted within the context of institutional practice patterns rather than as a generalizable trend.

In this study, procedural success was defined primarily as technical device deployment. Broader hemodynamic improvement and long-term clinical outcomes were not incorporated into the primary success definition, which may differ from criteria used in some prior studies. While lower than meta-analyses reporting 92%–98% success rates in preterm infants (12, 13, 20), our results align with single-center studies, such as Choi et al.’s 86.3% (27) success rate in patients under 6 months and Baspinar et al.’s 81% success rate in infants with a mean birth weight of 1700g, highlighting the influence sample characteristics such as of age and weight on outcomes. Although the chronological age at intervention ranged from 24 to 93 days, most patients were extremely premature at birth. Therefore, chronological age does not necessarily reflect physiological maturity or timing of ductal hemodynamic impact. In clinical practice, transcatheter closure is frequently performed after an initial period of medical management, stabilization, and adequate weight gain to ensure procedural safety. Thus, the timing observed in this cohort reflects individualized clinical decision-making rather than delayed treatment of hemodynamically significant PDA.

Device embolization was observed in 4 cases (7.7%). These findings are consistent with other single-center studies that reported device embolization, access-related vascular injury requiring surgical intervention, and device malpositioning as immediate complications occurring during transcatheter device closure procedures (28, 29). Interestingly, within the transcatheter cohort, smaller PDA diameter was associated with intraprocedural device embolization. Although larger PDAs were more frequently selected for catheter-based treatment, embolization risk may be influenced by procedural factors such as ductal morphology, shorter ductal length, and difficulty achieving stable device anchoring in smaller ducts. The reported ductal diameter reflects measurements documented in the medical records and may not consistently represent the minimal ductal diameter alone, as both echocardiographic and angiographic assessments were used in clinical practice. All procedures were performed using the Amplatzer Piccolo Occluder, and device sizing was individualized based on ductal morphology and minimal diameter in accordance with manufacturer recommendations and operator judgment. Given the small number of embolization events observed, this association should be interpreted cautiously and warrants further investigation in larger cohorts.

Approximately 21% of patients in this study had a residual shunt after transcatheter device closure procedure; however, in all cases, the shunt resolved by 6 months. Those who experienced residual shunt post-procedure had a larger mean PDA diameter compared to those who did not. The decrease or resolution of residual shunts has been documented in the literature, where both term and preterm patients undergoing device closure showed a decrease in the rate of residual shunt from immediately after the procedure to long-term follow-up (30, 31). Collectively, these findings suggest that while residual shunts may be present shortly after the procedure, they often resolve over time.

The high rate of tricuspid regurgitation in this cohort likely reflects functional regurgitation commonly observed in premature infants with elevated pulmonary pressures and volume overload secondary to hemodynamically significant PDA. The resolution of TR in a subset of patients following device closure may be explained by improved right ventricular loading conditions and reduction in pulmonary hypertension. However, due to the retrospective design, causal relationships cannot be definitively established.

Nonprocedural related death occurred in 9.6% of the entire sample. One of the deaths occurred in a patient who was born at term but had multiple complex comorbidities, including neurological conditions, craniofacial abnormality, and other congenital cardiac anomalies. The remaining deaths involved extremely premature patients. Although mortality was not directly attributed to the procedure, these patients represent a highly vulnerable population with extreme prematurity and multiple comorbidities. In such cases, distinguishing procedural effects from the natural course of severe neonatal illness can be challenging. While no direct procedural mortality was identified, indirect physiological stress related to intervention cannot be entirely excluded. Therefore, mortality findings should be interpreted within the broader context of prematurity-related morbidity.

Our secondary analysis involved determining the effect gestational age at birth and birthweight had on the outcomes. Analysis revealed a significant association between time of discharge and prematurity. We found a significant association between time to discharge and birthweight. As birthweight decreases, time to discharge increases. These findings highlight the significant impact of gestational age at birth and birthweight on patient outcomes, particularly time to discharge. The findings align with existing literature, which consistently demonstrates that prematurity and lower birthweight are associated with prolonged hospital stays (19, 32). This is likely due to the increased medical complexity and need for specialized care in premature and low-birthweight infants, who often require more time to achieve physiological stability and meet discharge criteria.

Regarding procedural outcomes, a significant difference between implant success and prematurity was found, where those who were premature were more likely to have implant success. It is expected that term patients would have a higher likelihood of implant success with fewer complications. However, due to the sample’s composition-80.0% of patients being premature, with 68.9% of the preterm group born before 31 weeks-the significant difference observed is likely attributable to the majority of the sample consisting of preterm patients. No statistical significance was found between birthweight and any of the procedural outcomes, which is most likely due to a small sample size.

### Study limitations

This study has several methodological limitations, including a relatively small sample size, which reduces the statistical power of the results and may limit the generalizability of the findings to larger populations. The retrospective design resulted in missing data for some variables across patient charts and limited access to detailed procedural documentation in some cases, including the exact mechanism of embolization resolution. Incomplete documentation of PDA subtype in a substantial proportion of patients further limits the ability to draw robust conclusions regarding morphological distribution. These data gaps restricted certain analyses and potentially impacted the comprehensiveness of our findings. The inclusion of patients with comorbidities and other cardiac lesions (except those requiring cardiac surgery) likely introduced confounding variables, potentially influencing the outcomes and limiting our ability to isolate the specific effects of the procedure. Furthermore, the absence of a control group significantly constrained the study’s comparative potential and overall interpretative scope.

The exclusion of patients requiring surgical intervention introduces potential selection bias, as these cases may represent more complex PDAs with different risk profiles. This may lead to overestimation of procedural success and safety. Therefore, our findings are most applicable to neonates considered suitable candidates for transcatheter closure and may not be generalizable to all PDA patients.

Lastly, as this was a retrospective study with predefined IRB-approved data variables, detailed procedural technical information, including vascular access approach and specific management of device malposition, was not systematically extracted, which limits procedural-level analysis.

## Conclusions

Transcatheter device closure demonstrates high efficacy for PDA management in preterm infants, with 100% ultimate success after repeat interventions. Larger PDA diameter and presence of murmur significantly predict the need for catheter-based treatment. While intraprocedural complications and residual shunts occur, most resolve spontaneously or with secondary intervention. Prematurity and lower birth weight significantly prolong hospitalization but do not preclude successful outcomes. Despite limitations related to sample size and retrospective design, these findings support the safety and effectiveness of transcatheter PDA closure in carefully selected neonatal populations, though larger prospective studies are needed.

## Data Availability

The original contributions presented in the study are included in the article/Supplementary Material, further inquiries can be directed to the corresponding authors.
